# The Psychological Distance and Climate Change: A Systematic Review on the Mitigation and Adaptation Behaviors

**DOI:** 10.3389/fpsyg.2020.568899

**Published:** 2020-11-19

**Authors:** Roberta Maiella, Pasquale La Malva, Daniela Marchetti, Elena Pomarico, Adolfo Di Crosta, Rocco Palumbo, Luca Cetara, Alberto Di Domenico, Maria Cristina Verrocchio

**Affiliations:** ^1^Department of Psychological, Health, and Territorial Sciences (DiSPUTer), G. d'Annunzio University of Chieti-Pescara, Chieti, Italy; ^2^Department of Neuroscience, Imaging, and Clinical Sciences, G. d'Annunzio University of Chieti-Pescara, Chieti, Italy; ^3^Eurac Research, Rome Office, Rome, Italy

**Keywords:** climate change, psychological distance, adaptation, mitigation, pro-environmental behavior, resilient behavior, environmental attitudes, construal level theory

## Abstract

**Background and Objective:** Currently, climate change represents an existential, physical, and psychological threat. Therefore, mitigation and adaptation actions and measures have become increasingly necessary to preserve individual and collective well-being. The psychological distance is one of the main psychological constructs that explains the most concrete or abstract perception of the objects and events surrounding people. The psychological distance is a multidimensional construct, and in accordance with the construal level theory (CLT), temporal, hypothetical, spatial, and social distance are considered the most critical dimensions. This systematic review aims to provide an update of the literature on the role of psychological distance in the commitment to engagement mitigation and adaptation attitudes toward climate change.

**Method:** The review was carried out following PRISMA guidelines and a systematic search was performed on PubMed, Psycinfo, Web of Science, Cochrane, and Scopus databases.

**Results:** Nineteen articles have been identified as being eligible for the final synthesis. Results showed, in general, that individuals have a higher propensity to perform pro-environmental and resilient behaviors against climate change when it is perceived as more proximal and concrete within the construct of psychological distance. However, not all studies show this result. Some studies showed that, despite people considering climate changes as real and tangible, they do not perform mitigation and adaptation behaviors. Other studies showed that people implement these behaviors despite perceiving climate changes as distal and abstract.

**Conclusions:** The current literature shows the existence of a relation among psychological distance and pro-environmental and resilient behaviors applied to climate change. For a deeper understanding of the conflicting results that emerged, more studies are necessary to explore the possible presence of further psychological variables involved in the relation within psychological distance, mitigation, and adaptation in environmental contexts.

## Introduction

The theory of the Construal Level explains that there is a relation among the psychological distance and response of people to a specific event (Liberman and Trope, 1998, 2003). The psychological distance is composed of four dimensions: spatial, social, temporal, and hypothetical (Liberman et al., 2007; Liberman and Trope, 2008). Each dimension is interrelated to the others (Fiedler et al., 2012), despite the lack of commonalities (Trope and Liberman, 2012).

The psychological distance is one of the main psychological constructs that explains the most concrete or abstract perception of the objects and events surrounding people. An object, or event, can be perceived as psychologically close or far away. When it is perceived as psychologically close, it is represented as being more concrete, while when it is perceived as psychologically far away, the representation is more abstract (McDonald et al., 2015). Therefore, psychological distance is linked with different construal's of objects and events (Trope and Liberman, 2010). While the constructs on a concrete level are focused on the details, those on a more abstract level are concentrated on the “big picture” (McDonald et al., 2015).

Psychological distance, therefore, could be involved in pro-environmental and resilient behavior**s** in relation to climate change. People are more likely to perceive climate change more concretely when they perceive it more closely and, as a result, there may be a growing willingness to engage in pro-environmental and resilient behaviors. Whereas, when people have a more abstract representation of the event it is because climate change is perceived as more distant (McDonald et al., 2015).

The spatial distance represents the physical distance toward an event. Therefore, people perceive worsening environmental conditions to be occurring in remote geographical areas (Gifford et al., 2009). People often tend to perceive more serious climate changes in developing areas and less severe where they live (Jamieson, 2005; Reser et al., 2012). This may happen because there is a tendency for people to detach themselves from information that could increase fear (Shepherd and Kay, 2012). People tend to see the positive aspect of climate change when this is psychologically close to their place. On the contrary, individuals tend to incline to the negative aspect when climate change is psychologically distant.

The hypothetical distance relates to the probability of whether an event can happen, or not. It also relates to the certainty perceived regarding a future event (McDonald et al., 2015). The uncertainty of the climate change occurrence often leads to people not fully understanding the different climate change predictions and, therefore, incorrectly analyzing the probability of its occurrence (Budescu et al., 2012).

Regarding the temporal distance, although it is accepted that actual climate change is occurring and that it has consequences, people could perceive it as psychologically far because its related effects are far in the future. About climate change, Leiserowitz (2005) highlighted that people generally tend to perceive that impacts are happening now. Still, they tend to consider that in the future, the consequences of impacts will be more severe.

Finally, social distance explains how people can accept climate change and how they can socially ward off the phenomenon when the most severe threats are considered. The impacts of climate change are frequently perceived as more severe in developing countries and in more geographically distant zones (Gifford et al., 2009; Reser et al., 2012). Self-closeness to an event appears to be related to a greater concern. The way people perceive an event defines how “socially distant” they are from the situation. Indeed, with greater social distance, people can prepare to act as soon as possible.

Generally, when climate change is discussed, several interventions are considered to address it. These interventions are mainly represented by behaviors that minimize harm to the environment as much as possible, or even benefits it: pro-environmental behaviors (Steg and Vlek, 2009). Various types of environmentally significant behaviors have been identified in the literature. According to the value-belief-norm (VBN) theory of Stern et al. (1999) there are four environmentally significant behaviors: environmental activism, non-activist public-sphere behaviors (e.g., environmental citizenship, policy support), private-sphere environmentalism (e.g., consumer purchase behaviors, maintenance of household equipment, waste disposal behaviors), and behaviors in organizations (i.e., behaviors affecting organizational decisions).

Furthermore, all environmentally significant behaviors are influenced by four main causal variables: attitudinal (e.g., general environmentalist predisposition, behavior-specific norms and beliefs, perceived costs, and benefits of action), personal capabilities (e.g., financial resources, social status, behavior-specific knowledge, and skills), contextual factors (e.g., material costs and rewards, laws and regulations, available technology), and habit and routine (Stern, 2000). However, pro-environmental behaviors can be considered as interventions that positively modify the environment directly and indirectly. Other types of attitudes can be implemented, not to alter the surrounding environment, but to support the inevitable changes that the environment undergoes and to live safely with them: resilient behaviors. These types of behaviors do not act directly toward the environment, but toward the individual, by changing the lifestyle and attitudes in synergy with the changes in the surrounding environment. They are especially needed when the environment is already changing. As well as climate change, the change is already underway. There are two main intervention behaviors for climate change: mitigation and adaptation. Mitigation is the possible reduction of climate change, for example, through pro-environmental behavior such as the reduction of emissions, greenhouse gases, and the use of renewable energy and eco-sustainable products. Adaptation is the preparation and the coexistence with climate change by preventing and reducing the effects of its impact and by exploiting the possible opportunities that may derive from it. Adaptation attitudes are implemented through resilient behaviors such as different tourism strategies (e.g., more summer activities than winter activities in mountain locations) or changes in the cultivation of agricultural products more suitable for different temperatures and climate conditions due to climate change. However, resilient behavior can coincide with pro-environmental behavior as co-beneficial actions (Mayrhofer and Gupta, 2016). For example, growing a type of plant that is more resistant to insects that thrive in increasing temperatures, can lead to a reduction in the use of pesticides and, consequently, less air pollution.

The psychological distance can explain the commitment to engage pro-environmental and resilient behaviors and, consequently, a more significant engagement of mitigation and adaptation attitudes toward climate change. Perception, from a personal stance is essential because individuals are more probable to behave in favor of the environment, and/or respond resiliently to changes in the surrounding environment, when they perceive the problem of climate change as a difficulty that can have direct consequences for themselves (Lorenzoni and Pidgeon, 2006).

Implementing these types of strategies is often difficult, as a widespread commitment is required. In addition to the general commitment, motivation from a people's stance is also particularly important, because the adaptation responses, in particular, must start from the individuals themselves.

The present systematic review is intended to provide an upgrade of the literature on the role of the psychological distance in the commitment to engage in mitigation and adaptation attitudes toward climate change. The key query that pushed us toward this work addresses the complex and broad understanding of the link between psychological distance, pro-environmental and resilient behaviors, and the underlying reasons for the choice to engage, or not, in behaviors, intentions, and attitudes toward the environment.

## Methods

### Information Sources and Searches

A systematic review of the literature was performed following the guidelines for systematic reviews and meta-analysis (PRISMA) (Moher et al., 2009). An electronic research strategy was carried out to identify peer-reviewed articles, assessing the role of psychological distance in the commitment to mitigation and adaptation attitudes to climate change up to mid-November 2019.

The keywords used for the review of the literature were: “climate change” OR “global warming” AND “psychological distance” OR “temporal distance” OR “spatial distance” OR “social distance” OR “hypothetical distance” AND “adaptation” OR “mitigation” OR “adaptation AND mitigation” OR “pro-environmental behavior” OR “resilient behavior” OR “environmental attitudes” OR “sustainable behavior.”

PubMed, Psycinfo, Web of Science, Scopus, and the Cochrane Library were used as databases for the systematic search.

### Eligibility Criteria

Only papers written in English were considered and if they examined the role of psychological distance on pro-environmental and resilient behaviors applied to climate change.

We excluded papers that considered further psychological variables related to mitigation and adaptation behaviors in the context of climate change. We also excluded theses, books, book chapters, meta-analyses, and reviews. Regarding study design, both qualitative and quantitative studies were included.

### Analysis and Data Synthesis

The eligible studies described several results, also with regards to sample, design, and measures. In order to have a more complete evaluation, both qualitative and quantitative studies were included. The studies were categorized by comparing the sample and highlighted measures evaluated for each study and by summarizing the main results.

## Results

### Study Selection

**Figure 1:**
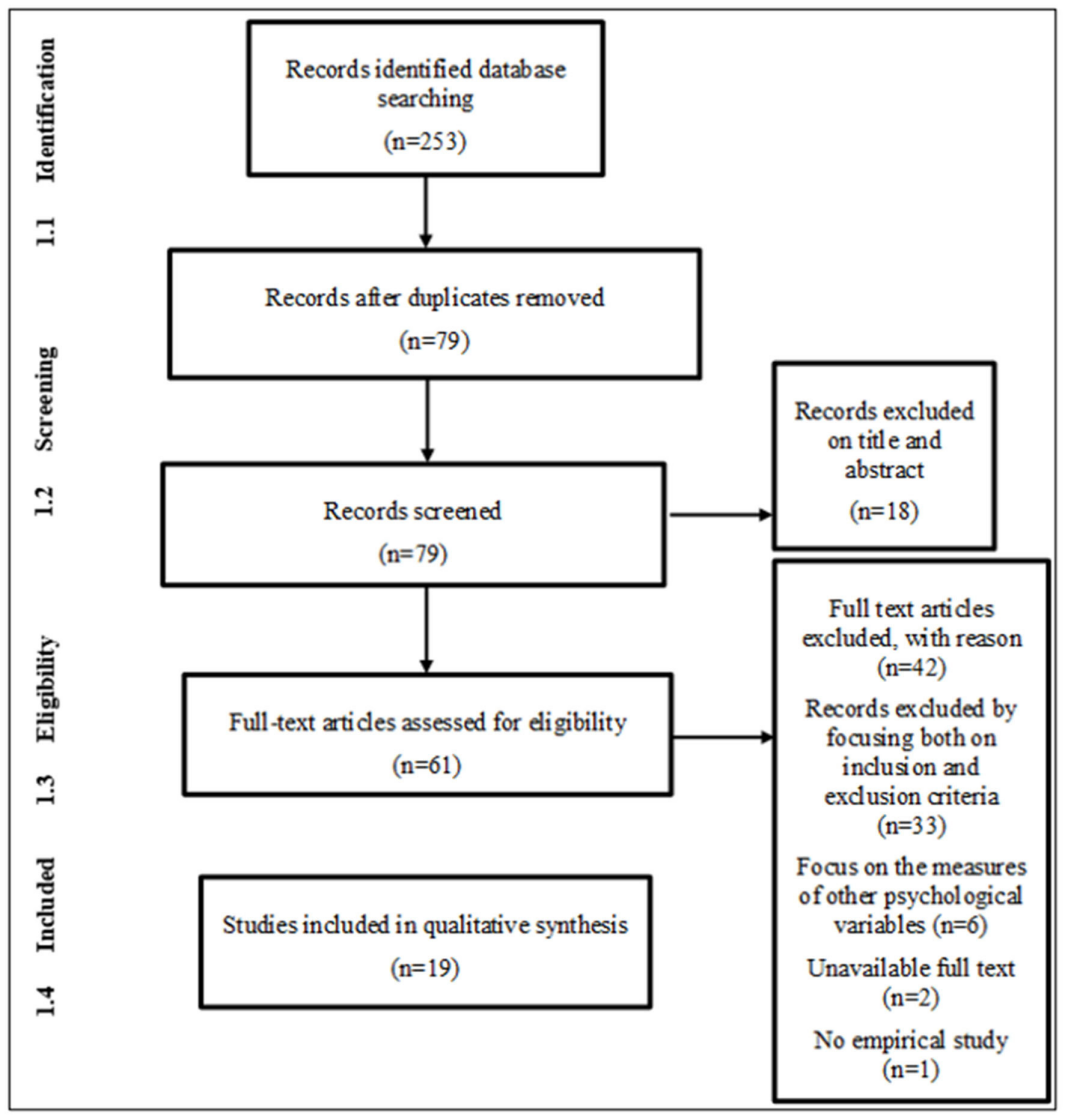
Flowchart of the systematic.

The search on databases initially produced 253 articles. Out of 253 articles, 79 were selected for the screening of the full text, and 42 were not included for other reasons (as shown in the PRISMA flowchart, Figure 1). Nineteen articles were considered as eligible and pertinent for the final qualitative synthesis. At an early phase of the research, 25 studies were thought to be eligible. Still, after the analysis of the full text, we considered it appropriate to exclude six studies that dealt with the construct of psychological distance in relation to other variables.

### Results of Studies

Regarding the psychological distance: 12 studies investigated the spatial distance; 12 studies investigated the temporal distance; 10 studies investigated the social distance, and 7 studies investigated the hypothetical distance (see Table 1).

**Table 1:** Features of included studies assessing psychological distance and climate change behavior.

**References “Title” (Sample's country)**	**Aims**	**Study type**	**Sample information**	**Type of psychological distance**	**Measure of psychological distance**	**Type of behaviors**	**Measure of behaviors**
Kyselá et al. (2019) “Attitudes to public spending on environmental risk reduction: the role of temporal and spatial distance” (Norway)	To find out whether a more positive attitude on delayed action and distant or near risk reduction of two different environmental problems is different.	Quantitative *ad hoc* questions	*N* = 1,714; 839 male, 875 female; mean age = NA (18+) 8 randomized groups with no statistically differences	Spatial Temporal	8 *ad hoc* questions (1 for each group) with 7-point Likert-type scale	Mitigation	The agreement to use public funds to reduce environmental risk in the same 8 *ad hoc* questions with 7-point Likert-type scale
Wang et al. (2019) “Climate change from a distance: an analysis of construal level and psychological distance from climate change” (Australia)	To understand the possible link between perceived psychological distance, construal level, and support for climate action.	Quantitative 3 Studies S1: Survey S2: Survey S3: Survey + Task	S1: *N* = 218; 104 male, 114 female; mean age = 47.35 (18–84) S2: *N* = 216; 111 male, 105 female; mean age = 43.48 (18–79) S3: *N* = 320; 122 male, 198 female; mean age = 20.83 (17–68); 7 randomized groups	S1: All four dimensions + Construal level S2: All four dimensions + Construal level S3: Temporal + Construal level	S1: Psychological distance 1: 18-item measure with 5-point Likert-type scale; Psychological distance 2: NP-item measure with continuous sliding scale (0–100) + Environmental behavioral identification form (BFI-E); Response category width (RCW) S2: Psychological distance 1; Psychological distance + Environmental behavioral identification form (BFI-E); General behavioral identification form (BFI-G); Response category width (RCW) S3: Short-form of the Psychological distance 1 + Augmented version of the Behavioral identification form (BFI); Response category width (RCW)	Mitigation Adaptation	S1: Individual pro-environmental behavior; Community-level pro-environmental behavior (policy choice) S2: Individual pro-environmental behavior; Community-level pro-environmental behavior (policy choice) S3: Pro-environmental behavior (survey); Donation behavior (task)
Schuldt et al. (2018) “Does reduced psychological distance increase climate engagement? On the limits of localizing climate change” (USA)	To explore whether exposure to proximal (vs. distal) visual cues in term of spatial distance would lead to increased support for climate change-mitigation policies.	Quantitative Task + Survey	*N* = 251; 103 male, 148 female; mean age = 36 (NA)	Spatial + Construal level	Distance judgment (task); Attention check (1 multiple-choice question); Construal level (video description)	Mitigation Adaptation	Policy support: 12-item measure with 10-point Likert-type scale
Carmi and Kimhi (2015) “Further than the eye can see: psychological distance and perception of environmental threats” (Israel)	To show that individual differences in psychological distance determine the interpersonal differences in the perception of their severity, the level of environmental emotions, and the willingness to sacrifice for the environment.	Quantitative Survey	*N* = 305; 153 male, 152 female; mean age = 25 (NA)	Social Temporal Hypothetical	Social distance: 1-item measure with 5-point Likert-type scale Temporal distance: 1-item measure with 5-point Likert-type scale Hypotheticality: 1-item measure with 5-point Likert-type scale	Mitigation	Willingness to sacrifice scale: 6-item measure with 5-point Likert-type scale
Haden et al. (2012) “Global and local concerns: what attitudes and beliefs motivate farmers to mitigate and adapt to climate change?” (California)	To show that global beliefs and concerns about climate promote farmers mitigation behavior, while psychologically proximate concerns for local climate impacts will promote farmers adaptation behavior.	Quantitative Survey	*N* = 162; NA male, NA female; mean age = NA (NA)	Temporal + Construal level	Perceived change in local climate: 2-item measure with 3-point Likert-type scale; Future local water availability concerns: 3-item measure with 4-point Likert-type scale; Future local temperature concerns: 3-item measure with 4-point Likert-type scale; Global climate change belief and concerns: 5-item measure with 5-point Likert-type scale	Mitigation Adaptation	Energy and N efficiency practices: 4-item measure with 5-point Likert-type scale; New irrigation practices: 3-item measure with 5-point Likert-type scale; New cropping practices: 3-item measure with 5-point Likert-type scale
Brügger et al. (2015a)“Hand in hand: public endorsement of climate change mitigation and adaptation”(UK and Switzerland)	To explore the relationship between mitigation and adaptation actions by examining the correlations between different types of mitigation and adaptation and by investigating people's motives to mitigate and to adapt.	Quantitative 2 Studies S1: Survey S2: Survey	S1: *N* = 612; 280 male, 254 female, 78 no report gender; mean age = 39.3 (16–83) S2: *N* = 309; 159 male, 150 female; mean age = 36.6 (19–81)	S1: Spatial, Social S2: Spatial	S1: Risk perception (spatial): 7-item measure with 2 levels (proximal vs. distal); Support for mitigation/adaptation policies and personal behavioral intentions to adapt/mitigate (indirect social) S2: Risk perception (spatial) 7-item measure with 2 levels (proximal vs. distal)	Mitigation Adaptation	S1: Support for mitigation policies with 14 propositions; Support for pro-active adaptation policies with 15-item; People's future intentions to engage in behaviors to mitigate with 10 actions; Personal behavioral intentions to adapt with 8 actions S2: Support for mitigation policies with 14 propositions; Support for pro-active adaptation policies with 15-item
Rickard et al. (2016) “Here and now, there and then: How “departure dates” influence climate change engagement” (New York State and Singapore)	To explore how manipulating temporal and spatial distance in the context of climate change messaging about “departure dates” can influence policy support, risk perception, and affect.	Quantitative Survey	*N* = 376; 2 groups = New York State and Singapore N (New York State) = 193; 120 male, 73 female; mean age = 19.55 (NA) N (Singapore) = 183; 53 male, 130 female; mean age = 22.44 (NA)	Spatial Temporal	6 Messages types (short stories) with 3 temporal distance (2020, 2047, or 2066) and 2 spatial distance (New York City vs. Singapore)	Mitigation Adaptation	Policy support: 12-item measure with 10-point Likert-type scale
Niles et al. (2015) “How limiting factors drive agricultural adaptation to climate change” (New Zealand: Marlborough and Hawke's Bay)	To assess how farmers' past climate experiences influence their concern for future climatic limiting factors (water and temperature) and in turn, their likelihood to adopt adaptation behaviors.	Qualitative Quantitative Interviews + Survey	*N* = 490; 2 groups = Marlborough vs. Hawke's Bay N (Marlborough) = 177; NA male, NA female; mean age = NA (NA) N (Hawke's Bay) = 313; NA male, NA female; mean age = NA (NA)	Spatial	Local water concerns: 5-item measure with 4-point Likert-type scale; Local temperature concerns: 4-item measure with 4-point Likert-type scale; Global climate change concerns: 5-item measure with 5-point Likert-type scale	Adaptation	Climate change adaptation practices: 6-item measure with 6-point Likert-type scale
Brügger et al. (2016) “Proximising climate change reconsidered: A construal level theory perspective” (UK)	To reconsider the widespread belief that focusing on proximal (vs. distant) impacts of climate change should directly increase people's motivation to support mitigation and adaptation actions.	Quantitative Survey	*N* = 80; 14 male, 66 female; mean age = 20.6 (18–50)	Spatial + Construal level	Perceived risk: 7-item measure with 2 levels (proximal vs. distal) with 5-point Likert-type scale; High-construal level skepticism: 6-item with 5-point Likert-type scale	Adaptation Mitigation	Support for mitigation policies: 11-item measure with 5-point Likert-type scale; Personal intentions to mitigate: 10-item with 5-point Likert-type scale; Support for adaptation policies: 12-item measure with 5-point Likert-type scale; Personal intentions to adapt: 9-item with 5-point Likert-type scale
Milfont et al. (2014) “Proximity to coast is linked to climate change belief” (New Zealand)	To observe the distance to coast effect in a sample of a coastal nation with the measure climate change belief and support for government action to regulate emissions.	Quantitative Survey	*N* = 5,815; 2,328 male, 3,487 female; mean age = NA (NA)	Spatial	Geographic and regional information (based on the smallest geographical units in the census)	Mitigation	Support for emissions regulation: 1-item measure with 7-point Likert-type scale
de Guttry et al. (2019) “Situating climate change: Psychological distances as tool to understand the multifaceted dimensions of climate change meanings” (Germany)	To analyze how local and global, past, future and present, and social and individual dimensions of climate change interact in people's framings of climate change.	Qualitative Semi-structured interview	*N* = 36; NA male, NA female; mean age = NA (NA)	All four dimensions	Global phenomenon; Local phenomenon; Uncertain phenomenon; Issue of future; Anthropogenically-driven phenomenon	Mitigation Adaptation	Issue of materialization; political issue
Chen (2019) “Social representations of climate change and pro-environmental behavior intentions in Taiwan” (Taiwan)	To examine how people attribute meanings to climate change using social representations theory to explore the relationships between the social representation viewpoints and the people's intentions to engage in pro-environmental behaviors.	Qualitative Quantitative Semi-structured questionnaire	*N* = 245; 111 male, 134 female; mean age = NA (NA)	All four dimensions	Psychological distance: 6-item measure with 7-point Likert-type scale	Mitigation	Pro-environmental behavior intentions: 5-item measure with 7-point Likert-type scale
Jones et al. (2017) “The future is now: reducing psychological distance to increase public engagement with climate change” (Australia)	To observe how the four dimensions of psychological distance would mediate the message framing effect on climate change concern, and that both psychological distance and climate change concern would mediate the message framing effect on mitigation behavior.	Quantitative Video + Survey	*N* = 333; 190 male, 143 female; mean age = NA (18+); 2 groups = proximal frame vs. distal frame; N (proximal frame) = 178; N (distal frame) = 155	All four dimensions	Psychological distance: 26-item measure (spatial = 6-item; temporal = 8-item; social = 5-item; hypothetical = 7-item) with 5-point Likert-type scale	Mitigation	Mitigation intentions: 7-item measure with 5-point Likert-type scale
Kim and Ahn (2019) “The moderating role of cultural background in temporal framing: focusing on climate change awareness advertising” (USA and South Korea)	To examine the effects of two temporal message frames in environmental advertising on attitude toward and intention to engage in the pro-environmental behavior.	Quantitative Image + Survey	*N* = 193; 27 male, 156 female; mean age: 20.82 (NA)	Temporal	Perceived temporal distance: 1-item measure with 7-point Likert-type scale	Mitigation	Attitude toward behavior with 7-point semantic-differential items; Behavioral intention: 3-item measure with 7-point Likert-type scale
Singh et al. (2017) “The perceived psychological distance of climate change impacts and its influence on support for adaptation policy” (United States)	To explore how an individual's perception of climate change impacts may influence their support for adaptation actions.	Quantitative Survey	*N* = 653; NA male, NA female; mean age = NA (NA)	All four dimensions	Psychological distance: 4-item with 7-point bi-polar Likert-type scales (if, when, where, and who)	Adaptation	Support for adaptation policies: 6-item measure with 5-point Likert-type scale
Spence et al. (2012) “The Psychological Distance of Climate Change” (Great Britain)	To provide an exploration of all dimensions of the psychological distance on climate change, and how the different dimensions of relate to each another.	Quantitative Survey	*N* = 1,822; 875 male, 947 female; mean age: NA (15+)	All four dimensions	Geographic distance: 2-item measure with 5-point Likert-type scale; Social distance: 2-item measure with 5-point Likert-type scale; Temporal distance: 1-item measure with 7-point Likert-type scale; Uncertainty/skepticism: 1-item measure with 6-point Likert-type scale and 4-item measure with 5-point Likert-type scale	Mitigation	Preparedness to act: 1-item measure with 5-point Likert-type scale
Busse and Menzel (2014) “The role of perceived socio spatial distance in adolescents' willingness to engage in pro-environmental behavior” (Germany)	To examine the effect of perceived social and spatial distance on adolescents' willingness to engage in pro-environmental behavior.	Quantitative 2 Surveys	*N* = 938; 2 groups = Germany vs. “Developing Country”; N (Germany) = 470; 209 male, 254 female, 7 unspecified; mean age = 16.35 (14–19) N (“developing country”) = 468, 208 male, 252 female, 8 unspecified; mean age = 17.56 (12–18)	Spatial Social	Egoistic awareness of consequences resulting from ecological problems: 3-item measure with 5-point Likert-type scale; Biospheric awareness consequences resulting from socio-economic problems: 3-item measure with 5-point Likert-type scale	Mitigation	Willingness to engage in pro-environmental behavior: 11-item measure with 5-point Likert-type scale
Griffioen et al. (2019) “Which construal level combinations generate the most effective interventions? A field experiment on energy conservation” (Netherlands)	To investigate which combinations of high and low construal level interventions are most effective on a target behavior, warm water use, as well as on related behaviors, such as electricity use.	Quantitative task + 2 Surveys (Pre-intervention and Post-intervention)	*N* = 197; 2 groups = April 2015 vs. September 2015; N (April 2015) = 91; 45 male, 46 female; mean age = 22.13 (NA) N (September 2015) = 106, 46 male, 60 female; mean age = 20.36 (NA)	Social + Construal level	“How vs. Why” Task (2 conditions: low vs. high construal level); Trait construal level with 10-item Behavior Identification Form test (BIF); The option to choose a gift (task with 2 conditions: low vs. high social distance)	Mitigation	Water behavior (Shower and Shower time): 6-item; Electricity behavior (Switching off and Appliance use): 6-item; Pro-environmental behavior (Recycling, Buying envir-friendly products and Eating meat): 6-item measure with 5-point frequency scale
Soliman et al. (2018) “Wrinkles in time and drops in the bucket: circumventing temporal and social barriers to pro environmental behavior” (Canada)	To provide empirical evidence on the temporal distance, pro-environmental behavior and social norms.	Quantitative Survey + Task	*N* = 147; 30 male, 117 female; mean age: 18.8 (17–25)	Temporal	Subjective temporal distance (2 conditions: close vs. distant) + 2 measures with 11-point Likert-type scale	Mitigation	Environmental behavior inventory: 13-item measure with 5-point Likert-type scale; Environmental Intentions: the same 13-item measure with 5-point Likert-type scale

*N, Sample sizes; NA, Not Available*.

The studies are included in Table 1 following the alphabetical order of the title of the paper. For each study we also reported the geographical area in which the study was carried out in. This is especially relevant because the impacts of climate change may differ between different geographical territories and may consequently be perceived differently. The description of results for each study is divided into three paragraphs based on environmental behavior: two studies explored the role of psychological distance in the commitment to adaptation behavior toward climate change (Niles et al., 2015; Singh et al., 2017); 10 studies focused on engagement in mitigation with reference to some dimensions of psychological distance and climate change (Spence et al., 2012; Busse and Menzel, 2014; Milfont et al., 2014; Carmi and Kimhi, 2015; Jones et al., 2017; Soliman et al., 2018; Chen et al., 2019; Griffioen et al., 2019; Kim and Ahn, 2019; Kyselá et al., 2019); seven studies analyzed the link between psychological distance and each dimension with mitigation and adaptation behaviors applied to climate change (Haden et al., 2012; Brügger et al., 2015a, 2016; Rickard et al., 2016; Schuldt et al., 2018; de Guttry et al., 2019; Wang et al., 2019).

#### Psychological Distance and Adaptation

Singh et al. (2017), using a simple mediation model, examined the force and direction of the link between the perceived dimension of psychological distance of climate change effects, the individuals' degree of concern for climate change effects, and the individual's support for adaptation policies. For each dimension (social, spatial, hypothetical), the direct effect of psychological distance on supporting adaptation policies has been significant except for temporal dimension, that has a significant indirect effect on policy support, as it is completely mediated by the degree of concern for climate change effects. Regarding relation between climate change impacts and support of climate adaptation policies, the results showed a significant total effect of psychological distance compared to hypothetical distance, and a significant overall negative effect (c') was detected for social and spatial distance. Considering the level of concern and perceived effectiveness of an adaptation approach, no significant effect of the temporal dimension of climate change impacts on support of the combined measure adaptation policies was found. For all dimensions of psychological distance (spatial, temporal, hypothetical, and social), response effectiveness was negatively correlated to both concern for climate change impacts and support for adaptation policy. People's perception of the effectiveness of an adaptation approach mitigates the effect of concern for climate change impacts on their degree of support for the combined measure of adaptation policies. When the psychology of distance is reduced and concern rises, the response efficacy may explain lower levels of support for adaptation measures.

Niles et al. (2015) connected the ecological principle of “Liebig's law of the minimum” with the psychological distance theory. In this study, limiting factors within a farm system were considered (water or temperature impacts). The study was conducted by surveying farmers from two regions of New Zealand: Hawke's Bay and Marlborough. The adoption of adaptation practices was influenced differently by the limiting factors between the two regions and their farm systems. The limiting factors differed between farm systems and regions, in relation to past climate changes, agro-ecological context, infrastructure, and adaptation capacity. In particular, water acted as a limiting factor in Hawke's Bay, while water and temperature acted as limiting factors in Marlborough. In general, the results showed that past climate experiences have not influenced global concerns (major psychological distance) about climate change and, therefore, they did not promote the adoption of adaptation behaviors. Instead, the climate adaptation behaviors have been conditioned primarily by a local pathway (minor psychological distance) where past experiences influenced local concerns on future climate change (see also Azadi et al., 2019).

#### Psychological Distance and Mitigation

Griffioen et al. (2019), considering the construal level theory and the social psychological distance, analyzed the interaction and the effects of different approaches (high vs. low construal and social level) implicated in interventions on pro-environmental behaviors. In particular, the authors assessed the use of electricity and warm water (usage time) in a sample of students residing in one-person apartments in an all-inclusive student housing facility and evaluated, through surveys, their perceived sustainability, environmental self-identity, and self-efficacy. Four experimental conditions were composed, two congruent (high construal and high social level or low construal and low social level), and two incongruent (high construal and low social level or low construal and high social level). Which experimental condition could promote mitigation behavior during the intervention period (6 weeks) was explored. In the high social distance condition, subjects who were in a high construal level condition (congruent), decreased the use of warm water more than those who were in a low construal level condition (incongruent). In the low social distance condition, no difference was found between high (incongruent) and low (congruent) construal level conditions. The results suggest that a high construal level approach is exclusively efficient when merged with another high construal level approach (social distance), while it is not efficient when merged with a low-level construal approach. In the latter case, the low construal level component could be considered the driving element for behavior, which may not be very efficient when aiming for pro-environmental behavior. Furthermore, social distance manipulation individually did not show significant differences in the use of warm water for both construal level conditions. Regarding the effect of the experimental conditions on the use of electricity, the results showed that the social distance manipulation, independent from the construal level manipulation, had a significant effect on electricity use. Those who were in a high social distance condition decreased electricity consumption more than those who were in a situation of a low social distance condition.

Kim and Ahn (2019) studied the relationship between temporal psychological distance and pro-environmental behaviors using a sample of college students in the U.S. and South Korea. In particular, this study analyzed the effects of an environmental ad explaining the distant future (i.e., end of the twenty-first century, high temporal distance) vs. near-future (i.e., next summer, low temporal distance) effects of climate change and the engagement commitment to use low consumption light bulbs (mitigation behavior). The authors used a model based on the theory of the constructive level and its relationship with climate change mitigation behavior, including the cultural background as a moderator. In particular, the interactions of four dependent variables were considered: perceived temporal distance (proximal vs. distal), perceived relevance, attitude for the pro-environmental behavior, and behavioral intention. The results showed that the perceived temporal distance had negative effects on attitude toward behavior and perceived relevance; subjects exhibited a more positive attitude toward behavior and higher perceived relevance when they perceived the future effects of climate change as temporally more proximal. Additionally, perceived relevance had a positive effect on attitude toward behavior, and attitude toward behavior positively influenced behavioral intention. Regarding the role of cultural background, when exposed to the distant-future frame, South Korean subjects were inclined to perceive the distant-future (high temporal distance) effects of climate change as more impending and personally important. They also reported a more positive demeanor for behavior and higher behavioral intention than U.S. subjects. Regarding the near future (low temporal distance) frame, though, no significant differences in the four dependent variables were found among U.S. and South Korean subjects. In short, a lower temporal distance level promoted a greater attitude toward climate change mitigation behavior, regardless of the geographical area.

Kyselá et al. (2019), investigated the stability of individuals' responses related to temporal and spatial characteristics of policy scenarios in the public funding condition on climate change or air pollution risk decrease. They also explored if political orientation conditions these effects for the two issues. The authors used a factorial survey experiment, carried out in Norway, to investigate temporal and spatial distance on environmental issues. The scenarios consisted of three characteristics: spatial scale, timing, and target risks (air pollution or climate change). The items of the survey investigated the decrease of climate change or air pollution in Norway or the world. The authors highlighted the effect of geographical distance in the scenarios (air pollution or climate change). The results showed that air pollution (a local issue) received a higher agreement regarding immediate public spending. On the contrary, climate change scenarios (perceived as more distant) received a higher agreement for delayed public spending. Little or no evidence has been found about the influence of political orientation.

Soliman et al. (2018), using an experimental methodology, provided empirical evidence for temporal distance, pro-environmental behavior, and social norms. The results showed that a given message could help consider the problem as subjectively current and stimulate a change of actions toward situations that could happen in a distant future. The findings showed that, when they were considered individually, neither subjective temporal proximity nor social norms encouraged ecologically sustainable behavior. When they were considered together, they increased both mitigation intentions and behaviors. The subjects that have received the most support to perceive objectively distant results as subjectively imminent have reported a greater willingness to commit in ecologically responsible behavior, and therefore, stated having implemented sustainable behavior in the weeks succeeding the study. Accordingly, believing that climate change is imminent is not enough to become in favor of the environment, as people need to believe that their efforts are also part of social norms.

Jones et al. (2017), through the construal level theory, studied if communication intervention, focused on the reduction of psychological distance from climate change, could increase people's commitment in mitigation behavior. The authors created two treatment conditions, a multimedia message framed to increase psychological distance (distal condition), and a message framed one to decrease psychological distance (proximal condition). To measure the concerns on climate change and individuals' intentions to commit to mitigation behavior, subjects were casually included in one of two conditions. Moreover, the authors observed whether the effects of the treatment frames on climate change concern and mitigation intentions were totally mediated by all psychological distance dimensions (spatial, temporal, hypothetical, and social). The results indicate that the spatial, temporal, hypothetical, and social dimensions, are all significantly positively associated with climate change concern and mitigation intentions. The treatment frame manipulation was significantly associated with geographic (spatial), social, and uncertainty (hypothetical) of psychological distance dimensions, except for temporal distance. The participants in the proximal condition (lower psychological distance level) showed high levels of concern for climate change and strong intentions to undertake climate change mitigation behaviors compared those in the distal condition. However, through more in-depth analysis, it emerged that only hypothetical and social distances played an essential role in mediating the effect of the message frame manipulation. Subjects in the proximal frame condition considered climate change as less unsure and with greater probability to impact people like themselves. Additionally, reductions in perceived uncertainty and social distance were related to more climate change concern and greater intentions to commit to mitigation activities. Reduction of psychological distance could be achieved through messages that highlight the proximal impacts contrary to the distal of climate change. At the same time, the impact of the message on the concern of climate change could promote mitigation behaviors.

Carmi and Kimhi (2015) measured how people perceive climate change and the relative threats as close to themselves (social distance), imminent (temporal distance), and certain (hypothetical distance) in order to explore the reasons behind the discrepancy between the environmental threat, public response, and individual behavior. In particular, the study considered two sources of threats: environmental damage, such as air and water pollution and waste, etc., and global warming. The results showed that both environmental and global warming threats had a positive relationship with the three dimensions of psychological distance. The analyses also revealed that psychological distance is a strong and significant predicting factor for the perception of the two environmental threats. Furthermore, the effect of the psychological distance from both threats was also measured in relationship to three variables: perceived severity, environmental emotion, and willingness to practice mitigation behavior. Environmental emotions and willingness to practice mitigation behavior, were negatively correlated with psychological distance. Therefore, a lower level of psychological distance encouraged individuals to express stronger emotions toward the environment and a greater willingness to adopt mitigation attitudes.

Busse and Menzel (2014) evaluated the impact of the perception of social and spatial psychological distance on the willingness of adolescents to commit to pro-environmental behavior (mitigation behavior) concerning the biospheric consequences related to climate change. The authors administered two different questionnaires to two samples that referred to either a socio-spatially or a national distant position: one relating to the country of residence of the participants (sample 1) and another relating to a developing country (sample 2). The results showed that willingness to commit to pro-environmental behavior was associated with all independent variables (awareness of consequences and perceived behavioral control) except for perceived helplessness which was correlated with willingness to commit to pro-environmental behavior in sample 1 only. Therefore, all variables were correlated with each other in sample 1. Regarding perceived helplessness, no correlation was found. In sample 2, perceived helplessness was not associated with whatever the variables that measured awareness of consequences were, while it was negatively associated with perceived behavioral control. The correlation of all the variables with the categorical variable codifying the socio-spatial reference, highlighted a significant correlation with all the independent variables. Therefore, the results showed that biospheric awareness of consequences was negatively associated with psychological distance but positively correlated with all other variables. A correlation between willingness to commit to pro-environmental behavior and socio-spatial distance was not found.

Milfont et al. (2014) investigated the relation between physical closeness and the real belief of climate change, starting from the assumption that closeness is related to direct experience or anticipation of climate change. The results demonstrated how important geographic location is in addressing climate change. Distance from the coast, and therefore lower concerns for floods, rising sea levels, and other issues related to living near the coast, significantly predicted the reduction of levels of beliefs in climate change together with an inferior level of support for carbon emissions regulation. Proximity to the coast, instead, seemed to raise confidence in climate change, as people living on the coast have different opportunities to experience climate change because they can suffer different impacts such as floods and storms and rising sea levels, which therefore require greater adaptation.

Spence et al. (2012) investigated all dimensions of psychological distance (proximal vs. distal: spatial, temporal, hypothetical and social) on climate change and their interaction, as well as concerns toward climate change and sustainable behavior intentions (mitigation behavior concerning the energy use reduction). A survey in a nationally representative British sample was administered. The results indicated that the association between different psychological distance dimensions were all positive and very significant. In particular, the results showed that climate change was perceived as spatially, temporally, and hypothetically proximal. Regarding social dimension, the results were mixed. Specifically, the respondents perceived climate change as socially distant. Impacts of climate change are probably greater when experienced by other people, but also socially proximal because these effects are considered to be the same for all people. Significant correlations between psychological distance, concern regarding climate change, and sustainable behavior intentions were also found. Moreover, through a mediation model analysis, this study demonstrated that when concern regarding climate change was integrated inside the analysis, it operated as a significant mediating variable, thus decreasing the direct relation between psychological distance and mitigation behavior concerning energy use reduction, and highlighted the importance of people's concern for climate change.

Chen (2019), based on the Social Representations Theory (SRT), developed a self-reported, semi-structured, questionnaire on social representations of climate change in Taiwan. Social representations theory refers to the social psychological processes implicated in the construction of everyday knowledge of risk and the common-sense comprehension of the emergence of contemporary risk concerns (Moscovici, 1973; Smith and Joffe, 2013). In this exploratory study, Structural Equation Modeling (SEM) was used to analyze the determinants of social representation factors that may influence the intentions of the public to commit to mitigation behavior. Through this analysis, four factors of social representation of climate change were extracted: emerging climate change risk, media coverage and influence, psychological distance, and pro-environmental behavior intentions. A positive significant correlation, of social representations of emerging climate change risk and media coverage and influence on pro-environmental behavior intentions, was found. In particular, the two factors (emerging climate change risk and media coverage and influence) were decisive because they predict the public's mitigation behavior. Regarding social representation of psychological distance, climate change was perceived as proximal. Nevertheless, no significant correlation between psychological distance and pro-environmental behavior intentions was found.

#### Psychological Distance, Adaptation, and Mitigation

de Guttry et al. (2019), across a qualitative method and 36 semi-structured interviews with inhabitants of North Frisia (Germany), analyzed the proximal and distant levels of all four psychological distance dimensions (spatial, temporal, hypothetical, and social) regarding materialization of climate change and political issue (indirectly, mitigation, and adaptation behaviors). The analysis of qualitative interviews presented an elaborated model. In particular, the perception of climate change regarding materialization of climate change and political issue oscillated among various, or also ambiguous, distances and proximities, sometimes combining them. These results have shown multifaceted climate change meanings, simple or binary descriptions, which usually explain the perception of climate change as a distant problem, thus demonstrating the complex nature of climate change consequences. For this reason, according to the authors, considering a mixed perception (distal and proximal) of the psychological distance dimensions could be the best approach to promote mitigation and adaptation behaviors.

Schuldt et al. (2018) conducted two experiments attempting to decrease the spatial psychological distance of climate change, to increase people's commitment and political support for mitigation and adaptation behaviors. In particular, the subjects performed a visual-spatial task that described the Maldives as relatively proximal or distal. Subsequently, the participants judged the geographic distance of the nation (both in experiment 1 and 2) and watched and summarized a video describing its climate vulnerabilities (only in experiment 2). The results showed an effect on spatial psychological distance in both experiments. The participants in the proximal condition defined the Maldives as geographically closer and depicted its climate effects using a more tangible language. However, the reduced psychological distance did not increase public commitment and policy support. Additionally, the reduction of the spatial psychological distance did not increase the commitment to mitigation and adaptation behaviors.

Brügger et al. (2016), taking into consideration the construct of fear and skepticism, highlighted how proximal (compared to distal) effects of climate change could raise the motivation of people to implement mitigation and adaptation practices (see also Brügger et al., 2015b). The results show that in subjects with a proximal perspective, fear was correlated with risk perception. Therefore, the more individuals fear climate change, the more they perceive it as a risk. On the contrary, fear and risk perception, in people with a distant mindset, were not systematically correlated with each other. The higher the degree of fear in a proximity dimension, the more people are supportive of mitigation policies. On the contrary, low levels of fear did not show an effect on mitigation policy support. Regarding adaptation, the result highlighted that skepticism is negatively correlated with support for adaptation policies in the distant condition except for participants who had a proximal mentality.

Rickard et al. (2016) analyzed how modifying temporal and spatial psychological distance associated with climate change messaging can influence policy support to tackle climate change (an indirect measure to engage mitigation and adaptation behaviors) across a survey with an integrated experiment. Two groups from distant geographic areas (New York State and Singapore) were recruited. The study consisted of evaluating a message describing the same negative effects of climate change that could occur in three different time frames (proximal=2020; distal=2047; more distal = 2066) either in New York State or Singapore (3 × 2 experimental design). A total of six experimental conditions were created and all subjects were included randomly into one of the six conditions. The authors investigated the subjects' political ideology (i.e., liberal vs. conservative) and included it in the analysis. Few results have been found confirming that exposure to these different messages has relevant effects on the perception related to climate. Despite this, it has been shown that exposure to different information is linked in relevant ways to the political orientation of individuals. In particular, regarding support for policies directed at mitigating and/or adapting to climate change, the analysis showed that exposure to different time frames and locations played a significant role in the preferences of U.S. conservatives. While both U.S. liberals and their counterparts in Singapore have shown more perseverance in their policy support according to condition. The main result showed that, in the U.S. sample, liberals reported higher scores for policy support with the message of proximal space distance (New York state) and with more distal time distance (2066). This score was significantly different from both conservatives and other conditions. The contrasting levels of the spatial (proximal) and temporal (distal) dimensions of distance psychology have promoted greater commitment to policy support to engage in mitigation and adaptation behaviors.

Wang et al. (2019) conducted three studies with Australian participants. Through two surveys (study 1 and 2); the authors investigated whether the construal level (abstract vs. concrete) and the psychological distance (proximal vs. distal) from climate change, predicted pro-environmental and resilient intentions (individual vs. community) and policy support (individual vs. community). With one experiment (study 3), they observed whether the manipulation of temporal psychological distance and the construal level could increase pro-environmental and resilient behaviors. Study 1 and 2 investigated the same variables regarding psychological distance, construal level, pro-environmental and adaptation behaviors, and policy support. The only difference was the scale used for the construal level measurement (BFI): a long version was used in study 2 (see Table 1). The results showed that: in study 1, the community level policy support was predicted by construal level, whereas inferior support for individual-level pro-environmental and resilient behaviors was predicted by perceived psychological distance to climate change. On the contrary, in study 2 psychological distance did not predict support for individual-level pro-environmental behaviors, but some demonstrations were found in relation to construal level. In study 3, in addition to compiling the same construal level scales and the same pro-environmental and resilient behaviors scales used in study 2, subjects were shown a video about the change in precipitation due to climate change in Western Australia–screenshots. The video could cover three different time intervals (past, present, and future) and was followed either by a question with a concrete construal level or by a question with an abstract construal level (how/why method; see Hansen and Trope, 2012; Soderberg et al., 2015). The results showed that, contrary to the construal level theory, construal level did not predict pro-environmental and resilient behaviors. Furthermore, a greater temporal distance from climate change was related to greater levels of commitment in mitigation and adaptation behaviors.

Brügger et al. (2015a), through online surveys in two European countries, investigated the relationship between social psychological factors and mitigation and adaptation behaviors. Risk perception based on spatial psychological distance (proximal vs. distal) was considered in regard to social psychological factors. Furthermore, willingness to tackle climate change, based on social psychological distance (individual actions vs. policy support), was considered in regard to mitigation and adaptation behaviors. First, the authors found that the willingness to engage mitigation and adaptation behaviors were strongly associated; individuals willing to engage in mitigation behavior were also willing to engage in adaptation behavior. The results showed that the distant risk perceptions predicted willingness of people to support mitigation and adaptation policies better than the proximal risk perceptions. Perception of proximal risk did not predict individual behavioral intentions to mitigate climate change. Both perception of proximal risk and distant risk, provided a contribution to the prediction of personal behavioral intentions. When solely considering individual adaptation behaviors, proximal risk perceptions was found to be a predictor.

Haden et al. (2012) analyzed whether past climate experiences and global and local concern of farmers, in relation to climate change, can affect their intent to use mitigation and adaptation behavior. The authors focused on a total of six agricultural practices in both, mitigation (buy fuel-efficient farm equipment, reduce electricity usage in farm operations, improve nitrogen use efficiency, adopt conservation tillage, install solar panels or wind turbines, and use biomass or biofuels for on-farm energy use) and adaptation (pump more groundwater, adopt drip or micro-sprinkler irrigation, concentrate surface water on less acreage, use drought-tolerant varieties, drill more wells, shift to less water-intensive crops). The factor analysis produced two types of dependent variables for mitigation (“energy and nitrogen efficiency practices” and “renewable energy technologies”) and two for adaptation (“new irrigation practices” and “new cropping practices”). The mediation variables involved local concern about the availability of water and change in temperature, and the belief for global climate change. The results showed an indirect effect on the two types of mitigation practices due to the perceived change in past water availability. Furthermore, mitigation practices were mediated exclusively through the beliefs and concerns on global climate change of farmers. Instead, for the two types of adaptation practices, local water concerns significantly influenced exclusively the new irrigation practices and played a mediating role in the effect of perceived change of water availability in the past. In general, in this study two assumptions were established. The first was that mitigation and adaptation behaviors are cognitively represented at different construal levels. In particular, a higher construal level leads to mitigation activities, whereas a lower construal level promotes adaptation activities. The second was that psychologically distant concerns were a determinant factor of mitigation activities while adaptation between these farmers was principally driven by their concern for local climate effects and therefore considered psychologically close.

## Discussion and Conclusion

The present study aimed to systematically examine published original research reports that analyze the role of psychological distance in the commitment to undertake mitigation and adaptation attitudes toward climate change. Although most of the results reported above showed that more pro-environmental and resilient behaviors are engaged through lower levels of psychological distance, the relationship between the two constructs is complex and still unclear. In fact, in some studies it seems that mitigation behaviors are mainly related to a high psychological distance (distal) and adaptation behaviors to a low psychological distance (proximal). For example, the study of Chen (2019) reported that proximal perception of psychological distance was not a determinant of the level of intention of the public to manage with climate change by committing to mitigation behaviors. However, further studies have described conflicting results. For example, Schuldt et al. (2018) showed in their results that there was no significant difference between the willingness to commit to mitigation and adaptation behaviors and spatial and social distance (proximal vs. distal). A similar result was observed by Busse and Menzel (2014) and Brügger et al. (2015a).

Furthermore, as shown in the analysis of the collected articles, there are several differences in the methodologies used for examining the role of psychological distance and pro-environmental and resilient behaviors. In fact, in the selected studies, different tools and measures were used for both psychological distance dimensions and mitigation and adaptation behaviors. This could explain the conflicting results. Additionally, only a few dimensions of psychological distance were observed in most of the selected studies. Specifically, in 12 studies, only one or two dimensions of psychological distance were measured, as already done in previous studies given the simplest manipulation and the best control of the observed variables (Nicolaij and Hendrickx, 2003; Spence and Pidgeon, 2010). However, to better analyze how the dimensions of psychological distance interreact, studies should investigate the effects of manipulating distance on all dimensions (spatial, social, hypothetical, and temporal) at the same time. All four dimensions of psychological distance were considered simultaneously only in six of the selected studies. This approach would allow us to detect optimal framing and to favor willingness to act on climate change through mitigation and adaptation behaviors (McDonald et al., 2015).

This review also considered studies that measured the construal level, a major construct related to psychological distance, which may have importance for how people react to demands of climate change. In five of the selected studies, the psychological distance and the construal levels (abstract vs. concrete) were related to pro-environmental and resilient behaviors. A previous study, which already investigated the constructive level linked to the will to act on climate change (Rabinovich et al., 2009; Sanna et al., 2010), demonstrated the existence of a connection between the two factors. However, Wang et al. (2019) observed no correlation between the levels of psychological distance and the construal level. In fact, when the psychological distance of climate change had lower scores, climate change was not perceived as more concrete. Although, high scores of pro-environmental and resilient behaviors were recorded equally. These results suggest the limited use of the construal level in predicting pro-environmental behaviors. Moreover, contrary to the expected results, the study of Griffioen et al. (2019) showed that a high social distance condition was correlated with high construal levels which had a greater effect on mitigation behaviors. For these reasons, the existence of the relation among psychological distance and construal levels is not always obvious, especially in the situation of climate change and related adaptation and mitigation behaviors.

Given the complexity of measuring the factors considered, further variables that modulate the relation among psychological distance and pro-environmental behaviors of mitigation and resilient behaviors of adaptation could be involved. Some studies, not considering in this review, have already considered other types of variables (psychological and non-psychological) and measured them in relation to psychological distance and pro-environmental and resilient behaviors (Sacchi et al., 2016; Steynor and Pasquini, 2019).

In conclusion, psychological distance and all its dimensions contributed to the commitment to adopt pro-environmental and resilient behaviors of mitigation and adaptation, respectively. Although, it is an important topic for the psychological and physical well-being of individuals and the well-being of the whole planet, the studies conducted are still limited. This is because the research field has only recently become interested in this topic. The aim of this systematic review is to offer a good scientific starting point for future studies aimed at exploring and deepening the link among psychological distance and mitigation and adaptation behaviors in the hope of encouraging them more and more.

## Data Availability Statement

All datasets generated for this study are included in the article.

## Author Contributions

DM, RP, LC, ADD, and MV contributed to the conception and project of this review. RM, PL, EP, and ADC conducted the literature search and wrote the first draft of the manuscript. RP, LC, DM, ADD, and MV revised the first draft of the manuscript. All authors participated to the subsequent drafting and rewriting of the manuscript and approved the final version of the manuscript.

## Conflict of Interest

The authors declare that the research was conducted in the absence of any commercial or financial relationships that could be construed as a potential conflict of interest.
